# Supplementary material for: Toxoplasma gondii infection induces early host cell cycle arrest and DNA damage in primary human host cells by a MYR1-dependent mechanism
Source: Commun Biol. 2024 Dec 16;7:1637. doi: 10.1038/s42003-024-07374-0 (PMC11649780; doi:10.1038/s42003-024-07374-0)
Supplement: Supplementary file 1 — Supplementary material [file 42003_2024_7374_MOESM1_ESM.pdf]

A

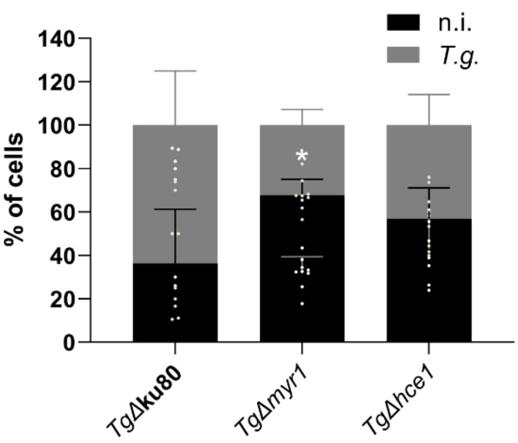

	% n.i. cells	% Infected cells
*Tg*RHΔ*ku80*	36.4 ± 25	63.6 ± 25
*Tg*RHΔ*myr1*	67.8 ± 7.3	32.2 ± 7.3 (*)
*Tg*RHΔ*hce1*	56.9 ± 14.2	43.1 ± 14.2

(*) Represent the statistical analysis between the n.i. cells.

(¥) Represent the statistical analysis between the infected cells.

B

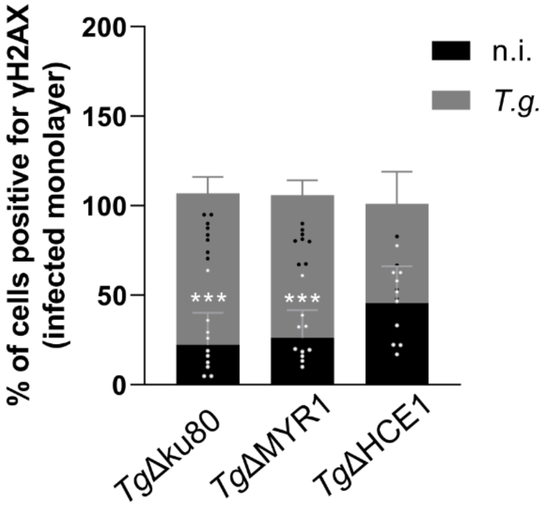

Figure S1: Infections rates in Figures 1 and 3 plots.
A) Percentage of infections in each parasite´s strain after 3 h p.i. (Figure 1-Sphase arrest) The table displays the average infection rate in each case.
B) Percentage of infections in cells positive to DNA damage at 3 h p.i. (Figure 3B)
All statistical analyses were a t-test with a $p<0.05$.

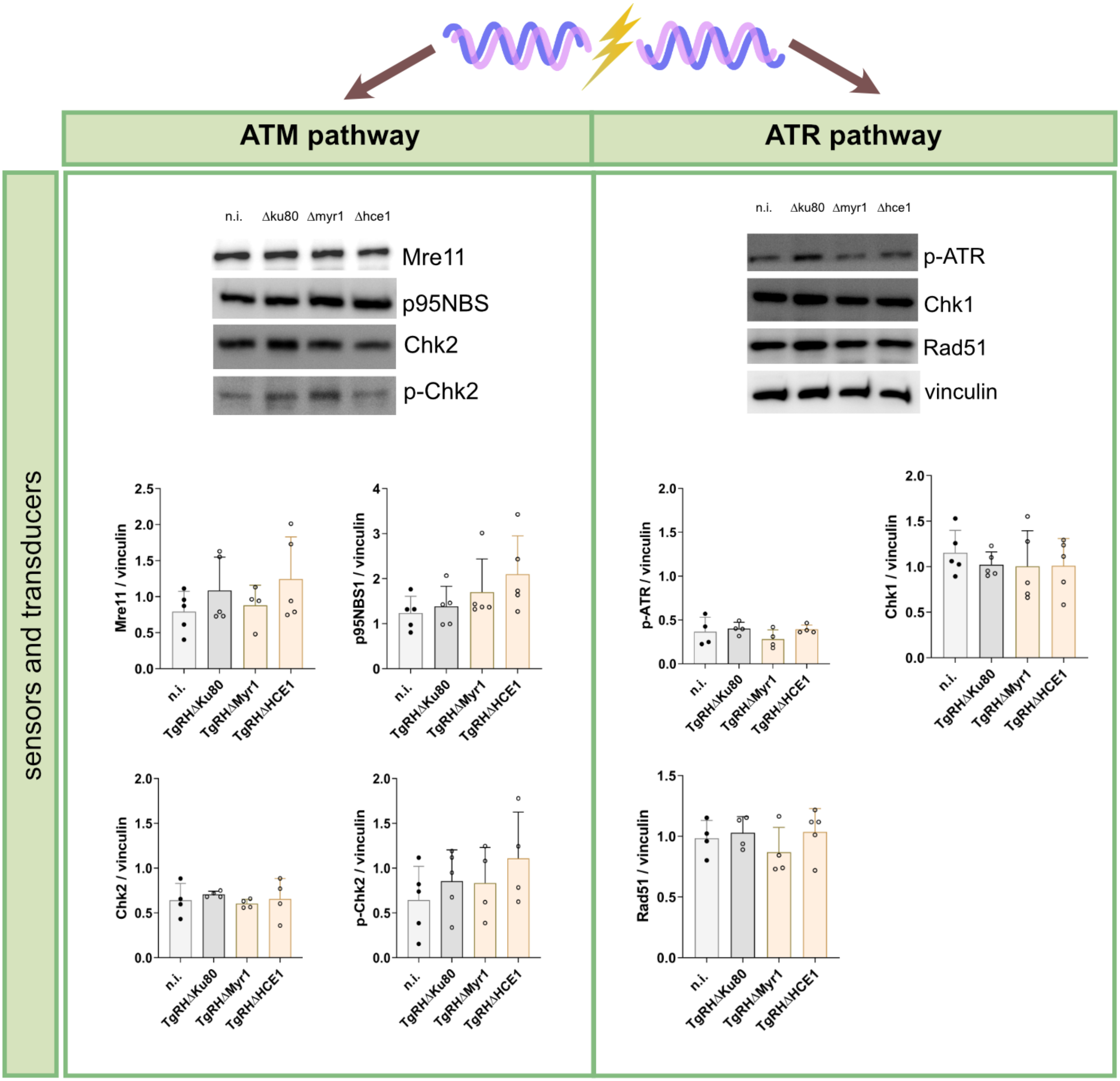

Figure S2: Proteins quantification by WB-based assay for ATM- or ATR-pathway.

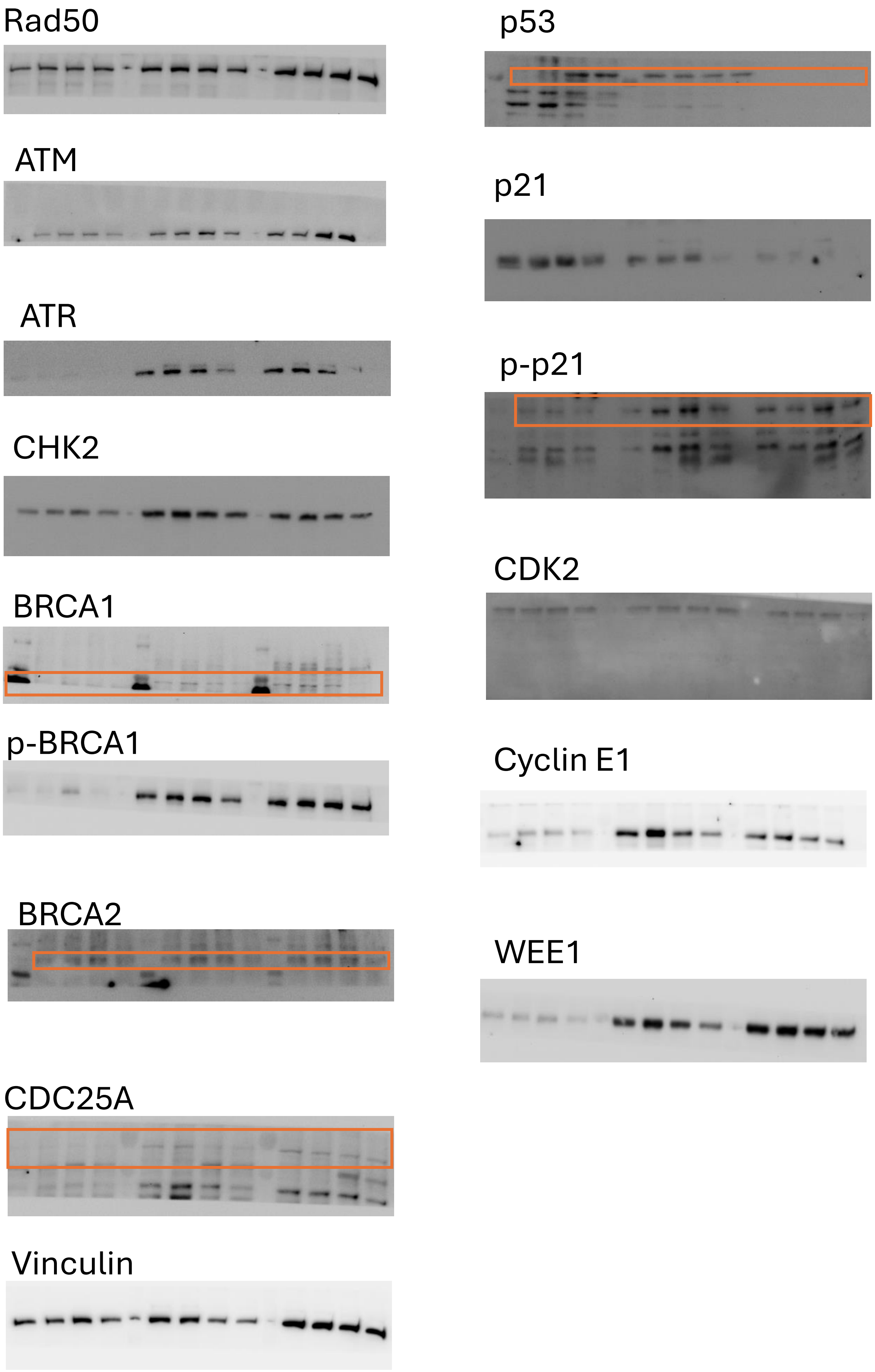

Figure S3: WB images Figure 5

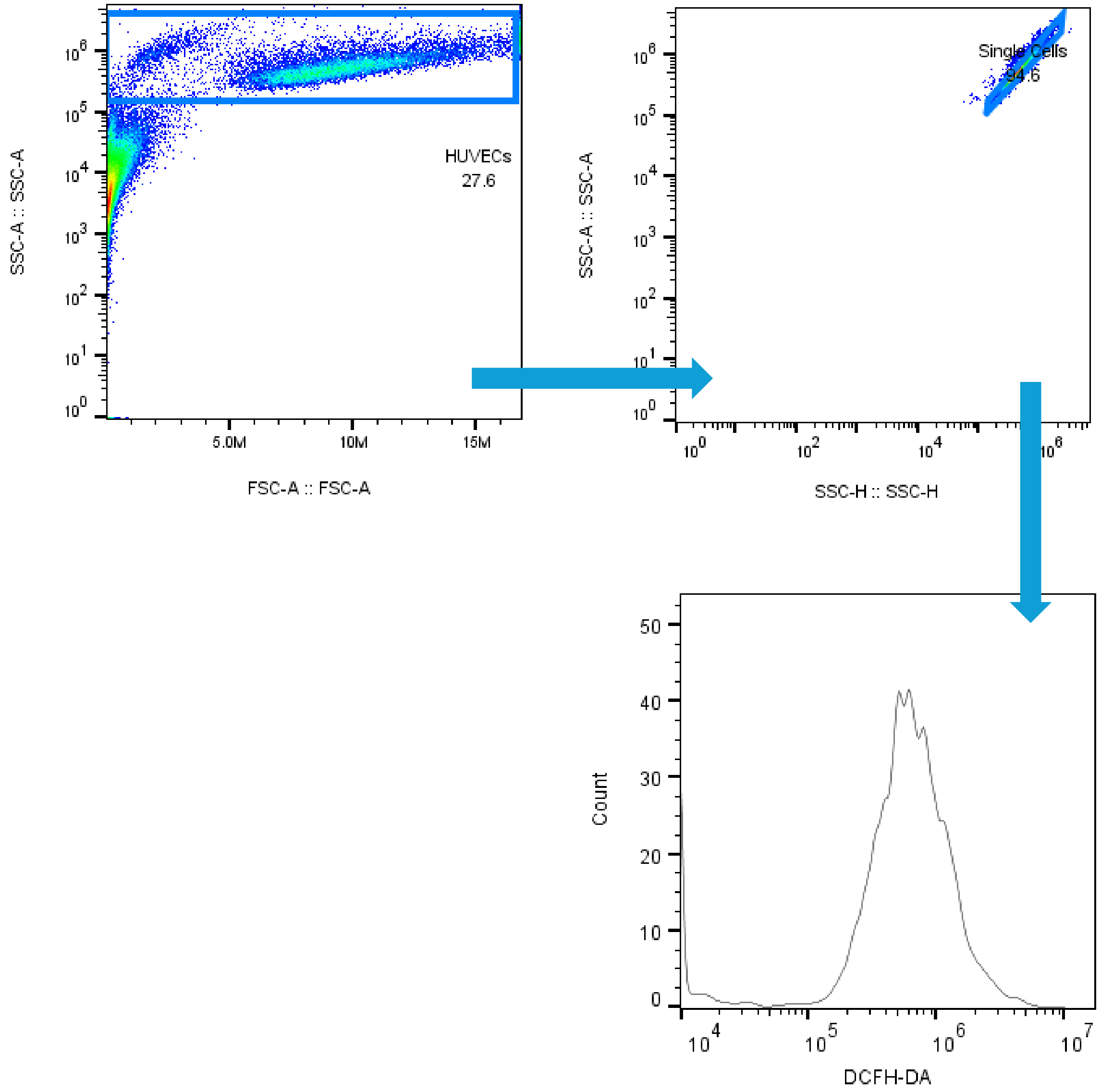

Figure S4: FACS gating strategy for Figure 6A
