# Supplementary material for: Toxoplasma gondii infection induces early host cell cycle arrest and DNA damage in primary human host cells by a MYR1-dependent mechanism
Source: Commun Biol. 2024 Dec 16;7:1637. doi: 10.1038/s42003-024-07374-0 (PMC11649780; doi:10.1038/s42003-024-07374-0)
Supplement: Supplementary file 2 — Description of Additional Supplementary Materials [file 42003_2024_7374_MOESM2_ESM.pdf]

Description of Additional Supplementary Files

File name: Supplementary Data 1

Description: Excel file with all graph raw data
