# Supplementary material for: Toxoplasma gondii infection induces early host cell cycle arrest and DNA damage in primary human host cells by a MYR1-dependent mechanism
Source: Commun Biol. 2024 Dec 16;7:1637. doi: 10.1038/s42003-024-07374-0 (PMC11649780; doi:10.1038/s42003-024-07374-0)
Supplement: Supplementary file 4 — Reporting Summary [file 42003_2024_7374_MOESM4_ESM.pdf]

nature portfolio

Corresponding author(s): Zahady D Velasquez

Last updated by author(s): Dec 2, 2024

Reporting Summary

Nature Portfolio wishes to improve the reproducibility of the work that we publish. This form provides structure for consistency and transparency in reporting. For further information on Nature Portfolio policies, see our Editorial Policies and the Editorial Policy Checklist.

Statistics

For all statistical analyses, confirm that the following items are present in the figure legend, table legend, main text, or Methods section.

n/a	Confirmed	
☐	☒	The exact sample size (*n*) for each experimental group/condition, given as a discrete number and unit of measurement
☐	☒	A statement on whether measurements were taken from distinct samples or whether the same sample was measured repeatedly
☐	☒	The statistical test(s) used AND whether they are one- or two-sided<br>*Only common tests should be described solely by name; describe more complex techniques in the Methods section.*
☐	☒	A description of all covariates tested
☐	☒	A description of any assumptions or corrections, such as tests of normality and adjustment for multiple comparisons
☐	☒	A full description of the statistical parameters including central tendency (e.g. means) or other basic estimates (e.g. regression coefficient) AND variation (e.g. standard deviation) or associated estimates of uncertainty (e.g. confidence intervals)
☐	☒	For null hypothesis testing, the test statistic (e.g. *F*, *t*, *r*) with confidence intervals, effect sizes, degrees of freedom and *P* value noted<br>*Give P values as exact values whenever suitable.*
☒	☐	For Bayesian analysis, information on the choice of priors and Markov chain Monte Carlo settings
☒	☐	For hierarchical and complex designs, identification of the appropriate level for tests and full reporting of outcomes
☒	☐	Estimates of effect sizes (e.g. Cohen's *d*, Pearson's *r*), indicating how they were calculated

Our web collection on statistics for biologists contains articles on many of the points above.

Software and code

Policy information about availability of computer code

Data collection	NIS Element software from Nikon.
Data analysis	Fiji open source software

For manuscripts utilizing custom algorithms or software that are central to the research but not yet described in published literature, software must be made available to editors and reviewers. We strongly encourage code deposition in a community repository (e.g. GitHub). See the Nature Portfolio guidelines for submitting code & software for further information.

Data

Policy information about availability of data

All manuscripts must include a data availability statement. This statement should provide the following information, where applicable:

- Accession codes, unique identifiers, or web links for publicly available datasets
- A description of any restrictions on data availability
- For clinical datasets or third party data, please ensure that the statement adheres to our policy

All relevant data are available from the authors upon request (zahady.velasquez@vetmed.uni-giessen.de). WB´s uncropped and unedited blot images are available in Supplementary Material file. Numerical source data for manuscript graphs are available in Supplementary Data_Excel file

Research involving human participants, their data, or biological material

Policy information about studies with human participants or human data. See also policy information about sex, gender (identity/presentation), and sexual orientation and race, ethnicity and racism.

Reporting on sex and gender	Not aplicable
Reporting on race, ethnicity, or other socially relevant groupings	Not aplicable
Population characteristics	Not aplicable
Recruitment	Not aplicable
Ethics oversight	Not aplicable

Note that full information on the approval of the study protocol must also be provided in the manuscript.

Field-specific reporting

Please select the one below that is the best fit for your research. If you are not sure, read the appropriate sections before making your selection.

☒ Life sciences ☐ Behavioural & social sciences ☐ Ecological, evolutionary & environmental sciences

For a reference copy of the document with all sections, see nature.com/documents/nr-reporting-summary-flat.pdf

Life sciences study design

All studies must disclose on these points even when the disclosure is negative.

Sample size	In our preliminary experiments, primary endothelial cells, HUVEC, showed a non-responsive donor in 10% of the cases. Therefore, quantitative experiments such as WB and ROS quantification were done in 6 biological donors.
Data exclusions	No data were excluded from the analysis
Replication	Only biological replication were done in experiments like WB and ROS quantification. Immunofluorescence was done using three biological donors and each one had three technical replicates
Randomization	Experiments were develop in six biological donor for WB-based protein quantification and ROS concentration. Immunofluorescence was done using three biological donors and each one had three technical replicates. All biological or technical replicates were performed at the same time in each set of experiments.
Blinding	Immunofluorescence quantification was done in images acquired by epifluorescence microscope. Every image was taken randomly to avoid bias

Reporting for specific materials, systems and methods

We require information from authors about some types of materials, experimental systems and methods used in many studies. Here, indicate whether each material, system or method listed is relevant to your study. If you are not sure if a list item applies to your research, read the appropriate section before selecting a response.

Materials & experimental systems

n/a	Involved in the study	
☐	☒	Antibodies
☐	☒	Eukaryotic cell lines
☒	☐	Palaeontology and archaeology
☒	☐	Animals and other organisms
☒	☐	Clinical data
☒	☐	Dual use research of concern
☒	☐	Plants

Methods

n/a	Involved in the study	
☒	☐	ChIP-seq
☐	☒	Flow cytometry
☒	☐	MRI-based neuroimaging

Antibodies

Antibodies used	This information is detailed in Table 1
Validation	All antibodies were described to recognize the human form of the protein. Therefore, they were directly tested in the primary endothelial cell (HUVEC) commercially acquired. We only included in the current study those that match the suggested size described by the manufacturer.

Eukaryotic cell lines

Policy information about cell lines and Sex and Gender in Research

Cell line source(s)	This information is detailed in Table 2. Al donors and catalogue number
Authentication	All are commercially acquired in PromoCell company (https://promocell.com/)
Mycoplasma contamination	All were mycoplasma negative tested by our Diagnostic service
Commonly misidentified lines (See ICLAC register)	None

Plants

Seed stocks	Not aplicable
Novel plant genotypes	Not aplicable
Authentication	Not aplicable

Flow Cytometry

Plots

Confirm that:

☒ The axis labels state the marker and fluorochrome used (e.g. CD4-FITC).

☒ The axis scales are clearly visible. Include numbers along axes only for bottom left plot of group (a 'group' is an analysis of identical markers).

☒ All plots are contour plots with outliers or pseudocolor plots.

☒ A numerical value for number of cells or percentage (with statistics) is provided.

Methodology

Sample preparation	This information is written in detail in lines 454-465 in the manuscript
Instrument	BD Accuri C6 Plus Flow Cytometer (Becton-Dickinson, Heidelberg, Germany)
Software	FlowJo v.10
Cell population abundance	Final analysis was done in 4500 cells.
Gating strategy	Cells were gated by shape and granularity. Then, the samples number was reduce to 4500 cells using "downsample" plugins. This reduces the number of events in a data matrix by generating a subpopulation containing cells distributed regularly or randomly throughout the selected parent population.

☒ Tick this box to confirm that a figure exemplifying the gating strategy is provided in the Supplementary Information.
